# Barriers to the Successful Health Care Transition of Patients with Kidney Disease: A Mixed-Methods Study on the Perspectives of Adult Nephrologists

**DOI:** 10.3390/children9060803

**Published:** 2022-05-30

**Authors:** Jenny Prüfe, Lars Pape, Martin Kreuzer

**Affiliations:** 1 Department of Pediatrics II, University Children’s Hospital Essen, Hufelandstr. 55, 45147 Essen, Germany; lars.pape@uk-essen.de (L.P.); martin.kreuzer@uk-essen.de (M.K.); 2 Psychosocial Service, University Children’s Hospital Essen, Hufelandstr. 55, 45147 Essen, Germany

**Keywords:** transition, barriers, nephrology, adult nephrologist, kidney transplantation

## Abstract

The transition from paediatric to adult-based health care is a challenging period bearing a high risk of medication nonadherence and transplant loss in adolescents and young adults after kidney transplantation. Successful transition asks for the cooperation of many, not least the adult physicians. Yet little is known about their thoughts and attitudes on the transition. We conducted a cross-sectional mixed-methods study, inviting all nephrologists registered with the German Society of Nephrology. A total of 119/1984 nephrologists answered an online survey, and 9 nephrologists participated in expert interviews on transition experiences and perceived barriers. Interviews were thematically analysed. Based on the results, 30 key statements were listed and returned to participants for a ranking of their relevance. The main themes extracted are (1) available resources, (2) patient-related factors, (3) qualification and (4) preparation of and cooperation with the paediatric setting. In conclusion, it became evident that successful transition faces multiple obstacles. At the least, it asks for time, staff, and money. Rigid structures in health care leave little room for addressing the specific needs of this small group of patients. Transition becomes a topic one wants to and is able to afford.

## 1. Introduction

The transition of adolescents and young adults with chronic health conditions from paediatric to adult-oriented health care is a challenging period both for patient and health care provider. It is associated with a high risk of negative health outcomes [1,2,3]. For instance, increased risk-taking behaviour and nonadherence in adolescents and young adults (AYA) with a kidney transplant (KTx) increase the risk of rejection and allograft loss [1,4,5]. Relative to age, the highest rates of KTx loss occur in patients aged 16–21 years [1,2,6]. Graft failure, however, requires a return to dialysis, which reduces quality of life, increases morbidity, shortens life expectancy, and leads to considerable additional health care expenses. 

Transition can be considered successful if it promotes the patients’ health competence, supports their psychosocial rehabilitation, and improves their self-determination, including their ability to make decisions and communicate about their care [2,6]. The overarching goal of transition is to enable patients to be as independent as possible and have the best possible quality of life. However, successful transition does not depend only on a structured process in paediatrics and good preparation of the patient. It also demands the thorough involvement of adult nephrologists, who unavoidably act as key stakeholders in the process [7,8]. Unfortunately, the transition from paediatric to adult care is often poorly organised. Communication between the paediatric system and the adult system is usually deficient, and many patients receive no care until an adult “problem”, such as pregnancy, forces them into the adult health care system [7,8,9]. Furthermore, it has been reported that orphan diseases and complex syndromic conditions in young adults were a challenge to transfer [9]. Paediatricians feel that adult nephrologists often are not trained to confidently deal with such diseases and dislike accepting those patients due to the extra time and effort needed in their care [9].

Few studies have investigated the transition-related activities, attitudes, and concerns of adult providers [7,8]. Even fewer have investigated the specific needs, concerns, and expectations of adult providers accepting the task of caring for chronically ill young adults [8,10,11,12,13]. Those existing studies were mainly based on patients with diabetes and cystic fibrosis, and data on patients with chronic kidney disease to date are almost nonexistent. The objective of this study was to fill this gap using qualitative and quantitative research methods aiming to elicit the needs, concerns, and expectations of adult nephrologists and to identify barriers to successful transition. 

## 2. Methods

The present research is part of the TRANSNephro project on transitioning paediatric renal transplant recipients into adult care [3]. The study has been registered at ISRCTN Registry (No. 22988897), and ethics approval was obtained from the ethics committee at Hanover Medical School on 4 July 2013.

In a first step, we conducted an online survey which was distributed to all nephrologists registered with the German Society of Nephrology (DGfN). The survey comprised multiple-choice questions aiming to collect information about the nephrologists’ general experiences in the treatment of formerly paediatric patients as well as on their specific needs for working with those patients. Open questions were added to further elaborate on the multiple-choice items. Additionally, participants were asked to indicate whether or not they were prepared to participate in a telephone interview. 

Semi-structured interview guidelines were developed based on the survey results, and research team discussions which included both paediatric and adult nephrologists. The interviews aimed to explore the participants’ personal experiences in caring for formerly paediatric patients and focused particularly on the challenges and obstacles related to this group of patients. In addition, details about staffing, patient numbers, and treatments were collected from the interview partners.

All interviews were conducted and transcribed verbatim by the same psychologist (JP). Transcripts were analysed using thematic analysis. Coding was performed independently by J.P. and M.K. and then discussed in the working group. Both the questionnaire and the interview guidelines are available online as Supplementary Data.

In a third step, we extracted 30 key statements from the interviews and sent them to all respondents from the initial survey who had provided their name and e-mail address and agreed to further contact. They were asked to rank the relevance of each statement for clinical practice on 4-point Likert scales. 

Statistical analysis was performed using Prism 6.0 (GraphPad Software, Inc., La Jolla, CA, USA). For analysis, items were ordered according to their mean rating and compared with each other using the Wilcoxon signed-rank test. A Bonferroni correction was used to adjust for multiple comparisons. The Friedman test was used to compare differences across different items. IBM SPSS Statistics 27.0 (IBM Deutschland GmbH, Ehningen, Germany) was used for reliability analysis.

Statistical significance was set at *p* < 0.05.

## 3. Results

### 3.1. First Survey Return Rate and Participant Data

**Figure 1:**
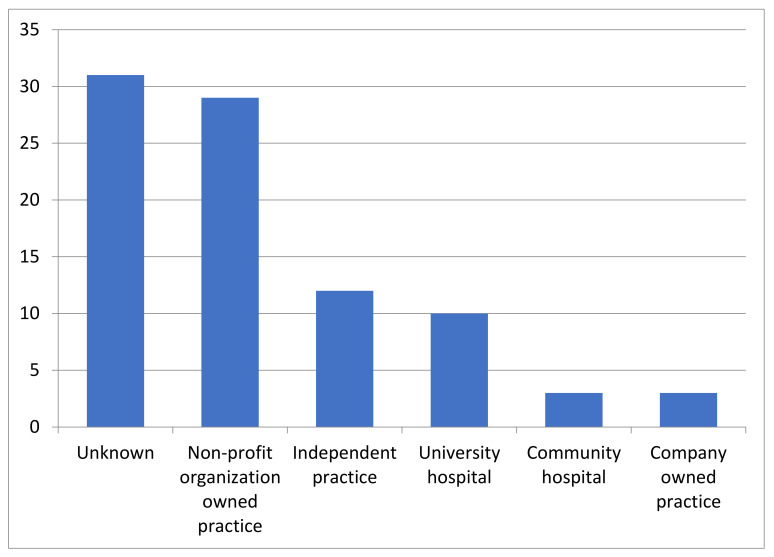
Affiliation of 119 survey participants.

The survey was sent to 1984 adult nephrologists via the DfGN and KfH mailing lists. Reminders were sent 2 and 6 weeks after initial distribution. A total 119 participants (6%) completed the survey, 57 respondents provided affiliation data and an e-mail address and agreed to further contact, and 44/119 (37%) participants declared their willingness to participate in an interview. The affiliation distribution of caregivers is displayed in Figure 1.

### 3.2. Survey Results

Of the 119 survey respondents, 88 nephrologists (74%) confirmed caring for young adults age 18–26 at the time of survey. The median number of patients of this age group under care was 4 (range 1 to 40); one third of participants cared for at least 10 young adult patients in their clinic. About 10% of participants did not provide patient numbers. Distribution of disease groups is displayed in Table 1. 

A total of 51 of the 88 nephrologists (58%) regarded caring for AYA patients challenging compared with their main patient population. Some of the nephrologists’ opinions on the challenges of this age group are given in Table 2.

About half of participants (40/88) articulated a need for further medical information relevant to treating AYA with childhood-onset chronic renal disease and most (77/88) wished for more information on social legislation topics for this group of patients. 

The question of who cares about the psychosocial concerns of the patients was answered by 47/88 as “myself/nephrologist”. Only 9/88 confirmed the existence of a social worker or psychologist at their centre, and 22/88 nephrologists indicated that they would not know whom to contact in case of psychosocial needs.

### 3.3. Interviews

Of the 44 (20%) nephrologists who were willing to participate in an interview, 9 were successfully contacted (university hospital n = 1, community hospital n = 1, independent practice n = 3, nonprofit organization n = 4). All but one of the interviewees regarded caring for formerly paediatric patients as challenging. Underlying factors could be grouped into: (1) available resources including finance and staffing, (2) patient-related factors including patient skills and social support, (3) qualifications including knowledge about adolescent health and rare disease, and (4) preparation of and cooperation with the paediatric setting.

### 3.4. Available Resources

All interviewees agreed that due to financial constraints it was difficult to provide the staff and time necessary to address the patients’ special needs. Several participants stated they knew colleagues who refused to treat formerly paediatric patients but also patients with developmental delay since the complex treatment of these patients was not sufficiently reimbursed. Particularly in the beginning of the doctor-patient relationship, time is needed to learn about another and build trust, yet not only a lack of time but also a high turnover of medical staff due to contract work impeded this development. Psychosocial staff appeared to be virtually non-existent because this part of the treatment was not reimbursed at all. Nonetheless, the nephrologists’ involvement in these patients’ care was regarded as essential. One participant remarked:

“If I don’t care for the patients’ psychosocial needs, no one does. Yet I am not a professional. My social support is as good or as bad as what I can provide as a father to my own children.”

(P4)

The same interviewee summarized:

“Transition is a difficult topic since you have to overcome various boundaries, including logistical and institutional constraints, for a small number of patients with very special needs without being sufficiently reimbursed. In the end it will always come down to the dedicated work of a few”.

(P4)

### 3.5. Patients Related Factors

All participants agreed that most of these patients lacked social and emotional maturity. This was said to result in teenage-like behaviour including a rather passive attitude, a lack of self-responsibility, denial of the disease and its challenges, oppositional behaviour, and not least nonadherence:

Patients first need to learn that appointments are not an annoying duty but personal care and focus on health and well-being. Many paediatric patients ask for rush-rush-treatment…quickly get the blood sample and please–no physician. Still they have to learn: focus on my body, my illness, my situation–care for myself and take the time.

(P1)

They have to learn that now they entered adulthood and have to take over responsibility. We can make them offers and give recommendations, but they have to decide themselves how to react to this.

(P6)

There was a perceived gap between the young adults’ cognitive and emotional development leading to them knowing what was important yet not being able to act accordingly:

“Those youngsters are not stupid. They can learn 300 new words at school but don’t know the name of their kidney disease. Something is really wrong here. This doesn’t match”.

(P4)

In addition to the medical demands, the nephrologists pointed out the social challenges which all tend to occur at the time of transfer, namely: completing school, finding a job, or moving out of the family home. This results in many shifts and changes at the same time, leading to excessive demands:

“One adds to another; maybe they become unemployed, can’t pay their mobile bills, start forgetting their appointments and then show up when it’s almost too late…”.

(P7)

In addition to the patients themselves, their families were perceived to play a role in the doctor-patient-interaction. Parents in particular were reported to have high expectations, which the nephrologists felt they could hardly meet. These expectations were paired with the parents’ anxiety that the new doctor might do something wrong, particularly when they did not prescribe the same medication as the paediatricians used to. Consequently, it was harder to build a trusting relationship.

### 3.6. Skills and Qualification

Most participants reported that the number of former paediatric patients transferred to adult-based nephrology was very low (“not more than 100 per year”) and that underlying paediatric diseases were rare compared with the diseases in the majority of their patients. Some interviewees observed that adult nephrologists lack training in paediatric diseases and adolescent medicine:

“Of course, the adult nephrologist does not know the underlying paediatric disease … well. Though, one could attend paediatric nephrology congresses–but I think that is unusual [regarding adult nephrologists]”.

(P6)

Many highlighted that care for patients with rare conditions depended on the individual commitment of the nephrologist. It was pointed out that some colleagues might lack interest in working with this group of patients. Some nephrologists who were integrated in larger multidisciplinary teams at hospital saw no need for additional training and reasoned that there were too few cases and that individual exchange was more fruitful. Others would appreciate educational offers (e.g., paediatric nephrology, adolescent medicine):

“We need–perhaps twice a year–training meetings at the transplant centre to communicate about post-transplant care and other relevant topics”.

(P6)

### 3.7. Former Paediatric Setting

It was remarked that formerly paediatric patients came from an environment of over-protection and “pampering”. Consequently, they had little chance to become aware of the seriousness of their situation and learn to take over responsibility. So, the “adult environment” could become demanding:

“Those patients are used to being sheltered and protected, and matters are told them in a roundabout way. For them the water here is just too cold to swim in. They are thrown in at the deep end yet they are poorly prepared”.

(P4)

All interviewees agreed that the interactions with paediatricians formed a key element of successful transition. Cooperation was regarded as a facilitator, whereas poor communication frequently led to difficulties in taking over and continuing the care for the young adult patient. 

Eight interviewees explicitly wished for a personal hand-over of the patients at least via phone and preferably a period of shared care lasting several months or years for a more gradual shift in care. Despite shared care being a preferred mode of organizing transition and transfer, only 2 respondents had regular experience with this. It was suggested that a lack of resources impeded paediatricians and adult nephrologists getting together:

“Getting together takes time but doesn’t bring money. Thus, it’s luxury.”

(P4)

The only interviewee who did not experience treating formerly paediatric patients as challenging summarized:

“I am only a stone’s throw away from the university hospital. I know the colleagues, I can call them, I regularly see them at meetings…and if patients transfer to my practice it’s a seamless transition”.

(P3)

### 3.8. Identifying Barriers and Ranking

We extracted 30 key-statements from the interview transcripts that we considered potentially relevant to the nephrological care for formerly paediatric renal patients. For reliability analysis, Cronbach’s alpha was calculated to assess the internal consistency of the 30-item scale. The internal consistency was high, with Cronbach’s alpha = 0.896. The corrected item-scale correlation coefficient is 0.49 ± 0.04 (median, 95% confidence interval).

The scale was sent to 57 nephrologists from the initial survey who had provided their e-mail address and agreed to further contact regarding the study. From those 57 nephrologists, 56 rated these statements with regard to their relevance. Items were ordered according to their mean rating and compared with each other using Wilcoxon signed-rank test. A Bonferroni correction was used to adjust for multiple comparisons, resulting in a significance level of *p* < 0.0017 and a Friedman test *p* value < 0.0001. Items with no significant difference in their mean ratings received the same ranking. The full ranking is given in Table 3.

Most frequently, the nephrologists regarded overprotective parents as a challenge not only to themselves and their medical work but also a factor that kept the young adults from becoming autonomous and self-responsible. This was followed by educational limitations and structural barriers such as a lack of training in adolescents’ medicine or insufficient resources to provide extra-medical support.

## 4. Discussion

To the best of our knowledge, this is the first in-depth analysis of the views, needs, and problems of adult nephrologists focusing on the transfer and further care of young adult patients with childhood-onset chronic kidney disease. We identified several barriers to successful health care transition. Relevant transition barriers covered 4 categories: (1) available resources including finance and (nonmedical) staffing, (2) patient-related factors including patient skills and social support, (3) qualifications, including knowledge about adolescent health and rare disease, and (4) preparation of and cooperation with the paediatric setting.

This aligns well with the findings of Peter et al. [7], who found in their survey of internists in different subspecialties that medical training, psychosocial issues, and financial support to care for patients with complex conditions were the dominating concerns. O’Conell et al. [11] raised the issue of the lack of communication between service providers in the care of disabled young adults. Although this topic was raised in the interviews, it was the point which was ranked lowest in the survey, indicating a satisfactory situation within our research population.

A question which arises is why, despite support by the medical society and several institutions, the response rate to our first survey was extremely low; Peter et al., e.g., received a 50% response rate [7]. One can only speculate: Lack of interest was one cause which was also mentioned by the interviewees. Another cause frequently mentioned was resource issues with surgeries being short of staff and spare time. Eventually, transition was not sufficiently within the scope of the nephrologists. At the medical centres of the 9 interviewees, former paediatric patients accounted for less than 2% of all patients, even at large centres.

A significant barrier in transition and ongoing care for former paediatric patients with childhood-onset renal disease is found in the health care system itself. This particularly includes financial resources, where time equals money (and vice versa). A lack of funds leads to a lack of staff, particularly nonmedical personnel. In consequence, some physicians perform the roles of psychologist and social worker as well, placing an additional burden on themselves. We assume, however, that in most cases, the nonmedical requirements of patients simply are not met. 

Most interviewees indicated their wish for a period of shared care with paediatricians to provide a gradual shift in care. Again, these resources are not budgeted in Germany, making side-by-side transition clinics rare exceptions in nephrology care.

Furthermore, paediatric nephrology centres have to improve transition and communication with adult caregivers. Recent studies show that the concept of transition is still being introduced to the adolescent patient too late [14,15]. Several tools were developed and tested to monitor transfer readiness, but their use in daily routine is scarce [15]. In addition, transfer age is frequently regulated and therefore not adapted to transfer readiness, and transfer of AYA without successful preparation is an avoidable extra burden for adult nephrologists. 

Speaking for themselves is a skill that many young adult patients have not acquired. With parents/caregivers advocating for the patient during all paediatric appointments, patient-doctor interactions can become challenging [16]. Interactions between health care professionals and patients in absence of caregivers should thus start in the paediatric nephrology setting, preferably years prior to transfer. It may be difficult for parents to increasingly hand over responsibility to the adolescent or young adult, particularly in times of change such as transfer [17]. It is therefore recommended that parents be involved in the early visits after transfer to build trust and confidence [16,17]. 

The most common age at transfer from paediatric to adult-based nephrology care in Europe, including Germany, is 18 years [6,14]. Psychosocial, behavioural, and anatomical development at that age is still ongoing [1,18]. The interval between 18 and 25 years of age is a socially defined developmental stage called “emerging adulthood” [1]. Even though young adults in this age group may appear physically mature, brain maturation is not complete until the end of this period. Increased risk-taking behaviour paired with an intense desire to be normal and the importance of peers may lead to nonadherence and a high risk for adverse outcomes [1,19]. Young adults strive for autonomy, which may conflict with overprotective parents and the sheltered environment of paediatric nephrology centres. This may lead to discrepancies between knowing what to do and doing what you know, especially in the presence of peers [20]. This is physiologic and cannot be altered by transition programmes or paediatric involvement prior to transfer. As a logical consequence, increased experience with adolescent medicine is crucial, and internal medicine residents should be appropriately trained during education [21]. The nephrologists ranked the limited specialized training in youth health and adolescent medicine as third highest on our list. So, either this has to be implemented in internal medicine education [17] or another approach may be advisable: pooling adolescents and young adults (AYA) with chronic kidney disease at specialised institutions (e.g., young adult clinics) [22,23] instead of caring for them in countless nephrologist practices scattered all over Germany. An integrated young adult clinic was introduced in Oxford in 2006 by Paul Harden that provided psychosocial care alongside tailored medical care for AYA after kidney transplantation [22]. The program was a success, and this concept is transferable to Germany if it is warranted by health policy and sufficiently funded.

## 5. Conclusions

Successful transition faces multiple obstacles. It is particularly the aspects of financial resources and staff qualification which needs to be addressed first. These will serve as the capital to better train patients in health literacy and competent self-care, as well as to build more efficient transition structures such as joint-transition clinics.

Although re-funding of transition medicine should be discussed with health insurance and policymakers, medical education needs to be reconsidered as well. Adult-specialist training in Germany does not require any paediatric rotation, but knowledge on adolescent health is needed to be prepared for a growing population of young adults with complex health care needs. 

As patients’ need for support is not limited to medical questions but includes psychosocial and developmental tasks, the roles of psychologists and social workers in a multidisciplinary setting need to be discussed and strengthened. 

What is presented in this research, however, is nothing more or less than the perspectives of a group of adult nephrologists. As a next step, gaining insight into patients’ and paediatricians’ perspectives is needed to complete the picture. 

## Figures and Tables

**Table 1:** Distribution of renal diseases (88 adult nephrologists currently caring for adolescents and young adults).

Disease Group	Number of Nephrologists
Chronic kidney disease stage 1–4	60/88
Haemodialysis	34/88
Peritoneal dialysis	10/88
Post-transplantation follow-up	48/88

**Table 2:** Frequency of categories and free text examples regarded as relevant challenges to transition by 88 adult nephrologists in a short online survey.

Special Needs or Problems (Category)	Relevant Issue for Participants	Examples (Free Text)
Medical	36/88	Adolescent medicine. Rare disease.
Social	64/88	Influence of peers. Risk behaviour.
Psychological	70/88	Noncompliance. Nonadherence. Lack of self-reliance. Lack of disease awareness.
Physician-patient relationship	46/88	Overprotective parents. Distrust of patient. Time consuming appointment.

**Table 3:** Mean and median Likert ratings and Wilcoxon rank order of stage 3 survey items (Bonferroni correction: *p* < 0.0017, Friedman test *p* < 0.0001).

Item	Category	Mean Likert Rating	Median Likert Rating	Wilcoxon Rank
1. Overprotective parents hamper medical treatment.	patient-related	3.25	4	22
2. It is difficult to involve parents without interfering with the development of patient autonomy.	patient-related	3.07	3	21
3. Adult nephrologists’ training in youth health and adolescent medicine is limited.	qualification	2.89	3	19
4. There is a lack of qualified staff to care for the nonmedical needs of patients.	resources	2.86	3	19
5. Difficulties at school or work hamper medical treatment.	patient-related	2.82	3	19
6. Adult nephrologists’ training in rare, syndromal, and congenital diseases is limited.	qualification	2.82	3	19
7. Financing structures and administrative regulations do not allow for coverage of nonmedical needs.	resources	2.79	3	18
8. Patients lack autonomy.	patient-related	2.75	3	18
9. The relative scarcity of paediatric renal conditions makes it difficult for adult nephrologists to gain experience in the field.	qualification	2.71	3	18
10. Patients are oblivious to the severity of their disease.	patient-related	2.64	3	18
11. Patients are overstrained by too many changes occurring at the same time (e.g., medical transition plus school, education, and independence from parents).	preparation & cooperation	2.75	3	17
12. Structures of integrated health care during transition (e.g., transition clinics, side-by-side consultations, alternating consultations) are missing.	preparation & cooperation	2.71	3	17
13. Patients lack the ability to sufficiently express their needs.	patient-related	2.64	3	17
14. Patients lack sense of responsibility.	patient-related	2.61	2,5	17
15. Patients and/or their families have exaggerated expectations regarding the work and time resources of the adult nephrologist.	patient-related + resources	2.64	2,5	16
16. Training (e.g., seminars, training courses) in paediatric nephrology for adult nephrologists is scarce.	qualification	2.54	3	16
17. Health care regulations do not allow sufficient time to adequately care for the complex medical needs of former paediatric patients.	resources	2.46	2	15
18. Former paediatric patients are more often nonadherent (medication, appointments) compared with my other patients.	Patient-related	2.43	3	15
19. Meeting the special needs of former paediatric patients is difficult.	resources	2.36	2	14
20. Access to paediatric patient records is difficult.	Preparation & cooperation	2.29	2	14
21. Patients lack emotional maturity.	Patient-related	2.29	2	14
22. Information beyond medical facts (e.g., social history, behavioural difficulties, substance abuse) is missing.	preparation & cooperation	2.25	2	13
23. Cooperation with other medical specialists is difficult in cases of syndromic or multimorbid patients.	preparation & cooperation	2.25	2	13
24. Patients are often insufficiently prepared for transfer.	preparation & cooperation	2.18	2	13
25. Former paediatric patients distrust me because I cannot prescribe some medications they are used to.	resources	2.14	2	12
26. The handing-over is insufficient: relevant data are lacking, provided too late, or not at all provided until requested.	preparation & cooperation	2.11	2	12
27. Patients encounter adult nephrologists with scepticism and refusal.	patient-related	2.11	2	11
28. Patients are not able to name their primary disease.	patient-related	1.96	2	11
29. High turnover of medical staff makes it difficult for patients to build trust.	resources	1.86	2	10
30. It is difficult to contact the paediatric nephrologists at university hospitals.	preparation & cooperation	1.61	1	8

## Supplementary Materials

The following are available online at https://www.mdpi.com/article/10.3390/children9060803/s1.
