# Supplementary material for: Barriers to the Successful Health Care Transition of Patients with Kidney Disease: A Mixed-Methods Study on the Perspectives of Adult Nephrologists
Source: Children (Basel). 2022 May 30;9(6):803. doi: 10.3390/children9060803 (PMC9221888; doi:10.3390/children9060803)
Supplement: Supplementary file 1 [file children-09-00803-s001.zip › Supp 1 online survey.pdf]

Online survey questions (translated from German)

1) Do you have patients age younger than 26 with congenital kidney disease or a kidney disease acquired before the age of 18?
2) How many of these have you taken on from paediatric care?
3) Please sort your formerly paediatric patients into the following groups:
   a. CKD 1-4
   b. Haemodialysis
   c. Peritoneal dialysis
   d. Transplant follow up
4) How do you experience treating these young patients as compared to your older ones?
   a. unproblematic
   b. challenging [open text field for comments]
5) Which specific questions or problems do occur in you working with formerly paediatric patients?
   a. medical (e.g. different treatment regimen, rare condition, etc.)
   b. social (e.g. questions re work, family planning, disability allowances, etc.)
   c. psychological (e.g. emotional challenges, delayed emotional development, adherence, etc.)
   d. doctor-patient-interaction (e.g. involving parents, independent disease management, etc.)
   e. others, namely… [text]
6) Who is taking over the psychosocial care?
   a. physician
   b. nurse
   c. psychosocial staff within the own practice
   d. psychosocial staff shared with other institutes
   e. independent / self-employed psychosocial staff
   f. other, namely… [text]
   g. unknown
7) Would you wish for more information re young people with CKD?
   a. medical information
   b. psychological information
   c. information re social law
   d. others, namely… [text]
   e. none
8) Would you be willing to participate in an expert-interview on transitioning young adults with a kidney transplant? If yes, please leave your name, centre, and Email-address
9) Is there anything else you would like to let us know?
