# Supplementary material for: Barriers to the Successful Health Care Transition of Patients with Kidney Disease: A Mixed-Methods Study on the Perspectives of Adult Nephrologists
Source: Children (Basel). 2022 May 30;9(6):803. doi: 10.3390/children9060803 (PMC9221888; doi:10.3390/children9060803)
Supplement: Supplementary file 1 [file children-09-00803-s001.zip › Supp 2 Interviewguideline.pdf]

Interview-Guideline (translated form German)

Data to be obtained before the interview:

- How many patients do you care for at your centre?
- What day / time do you usually see transplant patients for follow-up visits and lab work?
- What is your staff ratio (in full time equivalent)
- Do you have specific activities / offers which are special to your centre?
- Please suggest a date and time which would be convenient for you for a telephone interview.

Interview prompts

Prompt 1: Please tell me about your experiences re transition.

Prompt 2: Let us focus on the challenges…

(hint: in our online survey 2/3 of the participants considered caring for formerly paediatric patients as a challenge. How do you experience the situation? Are there differences between formerly paediatric and your common patients?
If challenges are reported: How do you meet / cope with these challenges?)

Prompt 3: Is there anything you feel lacking?

(hint: 2/3 of the survey respondents wish for more input on social, legal, psychological or specific medical information relevant to the care of young adults. What are themes which you consider important / interesting?

Prompt 4: How do you experience interdisciplinary work and cooperation with paediatricians? What do you think about it?

(hint: what would you wish for from your paediatric colleagues and their team? What would you wish for with regards to and from the patients? What would you wish for from your own team to work as you reckon would be best for young adult patients?
